# It Takes a Village: Microbial Communities Thrive through Interactions and Metabolic Handoffs

**DOI:** 10.1128/mSystems.00152-17

**Published:** 2018-03-13

**Authors:** Laura A. Hug, Rebecca Co

**Affiliations:** a Department of Biology, University of Waterloo, Waterloo, Ontario, Canada

**Keywords:** biogeochemistry, enrichment culture, environmental microbiology, metagenomics

## Abstract

An enduring theme in microbial ecology is the interdependence of microbial community members. Interactions between community members include provision of cofactors, establishment of redox gradients, and turnover of key nutrients to drive biogeochemical cycles. Pathways canonically conducted by isolated organisms in laboratory cultures are instead collective products of diverse and interchangeable microbes in the environment. Current sequence-based methods provide unprecedented access to uncultivated microorganisms, allowing prediction of previously cryptic roles in biogeochemical cycles and interactions within communities. A renewed focus on cultivation-based methods is required to test predictions derived from environmental sequence data sets and to address the exponential increase in genes lacking predicted functions. Characterization of enriched microbial consortia to annotate hypothetical proteins and identify previously unknown microbial functions can fundamentally change our understanding of biogeochemical cycles. As we gain understanding of microbial processes and interactions, our capacity to harness microbial activities to address anthropogenic impacts increases.

## Abstract (precis)

An enduring theme in microbial ecology is the interdependence of microbial community members. Interactions between community members include provision of cofactors, establishment of redox gradients, and turnover of key nutrients to drive biogeochemical cycles.

## PERSPECTIVE

Microorganisms represent the majority of life on earth, both in cell numbers and in diversity. They conduct critical ecosystem services, including biogeochemical cycling and contaminant transformations. Interactions between microbial community members are essential for maintenance of these ecosystem services. In most cases, microorganisms live within complex communities containing diverse populations, with key biogeochemical cycle steps distributed between community members. With significant heterogeneity across environments, characterization of microbial interactions is an ongoing endeavor within microbial ecology. High-throughput sequencing approaches, including total community DNA sequencing, or metagenomics, have provided a torrent of information on microbial distribution, diversity, and metabolic potential. This perspective argues that sequencing and enrichment cultivation together represent a powerful combination of new and old tools for examining microbial interactions and for identifying currently uncharacterized microbial activities.

High-throughput sequencing combined with reconstruction of high-quality draft metagenome-associated genomes (MAGs) provides detailed information on taxonomy, metabolic potential, and organism abundance. Where initial metagenome studies were able to reconstruct genomes for the most abundant organisms in low-diversity environments, such as acid mine drainage or the oligotrophic ocean, genome binning from metagenome sequencing now routinely results in tens to hundreds, and even thousands, of MAGs for organisms within environments as complex as sediments and soils (1–4). This explosion in draft genome sequences includes genomic information for lineages that are uncultivated: candidate phyla that represent new branches on the tree of life (5). Draft and complete genome sequences for small-celled, small-genome-containing organisms (bacterial candidate phylum radiation [CPR] and archaeal supergroup DPANN) clarify why these lineages have resisted cultivation: members of these groups do not encode complete pathways for synthesis of basic building blocks such as nucleic acids and amino acids and thus cannot survive independently from the other microbes sharing their environments (6, 7). Environmentally derived MAGs for these organisms indicate that symbiotic or commensal relationships within microbial communities, lifestyles where the organisms are heavily dependent on community interactions, are substantially more prevalent than previously thought (6, 7).

**FIG 1:**
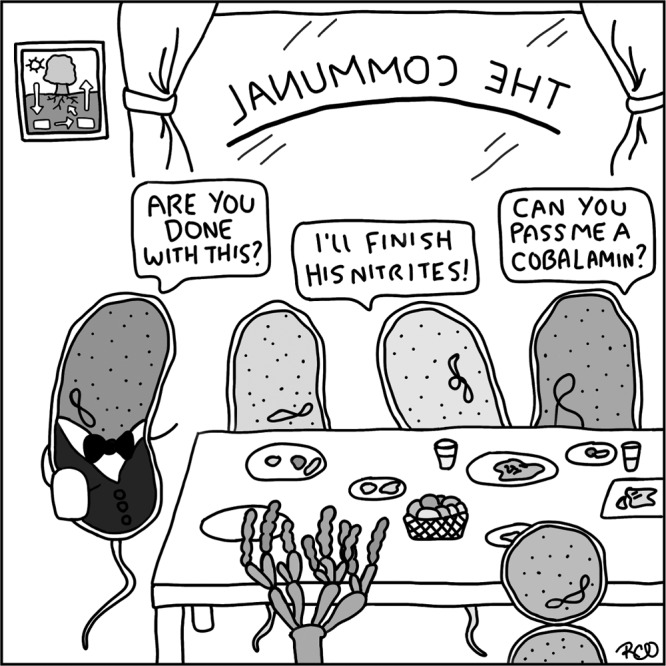
Microbes at a communal table depict interactions and handoffs occurring in the environment.

With hundreds or thousands of MAGs available from a single environment, organismal roles in nutrient cycling can now be examined from a whole-community perspective, within the framework of partial genomes for the majority of populations. Deep metagenomic sequencing of a terrestrial sediment system identified a striking number of broken pathways within the thousands of reconstructed microbial genomes (3). The vast majority of microorganisms with predicted roles in carbon, nitrogen, and sulfur cycling did not encode sequential pathway reactions within their genomes but instead are predicted to conduct fragmented or isolated functions within these cycles (3). Very few organisms with predicted roles in denitrification had the capacity to convert nitrate through to N_2_ (12 of 330, 3.6%). The majority of organisms implicated in denitrification contained a single pathway reaction encoded on their genomes, with the complete pathway dispersed across the microbial community. These trends held true for sulfur cycling as well, with the complete pathway from sulfide to sulfate present in only 3% of genomes for the organisms with predicted roles in sulfur oxidation, with single pathway reactions more commonly identified (3). Examinations of denitrification capacity across 652 microbial genomes from diverse environments identified truncated and partial denitrification pathways with various cooccurrences of the genes across microbial groups, further indication that denitrification is a highly modular pathway and that N_2_ formation is dependent on a functionally diverse community in many environments (8). From these genome-based studies, there is a growing awareness of the metabolic handoffs occurring within microbial communities (Fig. 1), alongside an expansion of the taxonomic groups implicated in these pathways. Metatranscriptomic and metaproteomic analyses are needed to clarify the predicted interactions and to determine which organisms are active within these cycles.

The current biggest challenge in environmental microbiology is annotating the thousands of hypothetical proteins identified from each new genome and metagenome—the known unknowns. Reconstructed genomes can have upward of 40 to 50% of their genes as predicted open reading frames with no known function (6). The databases of hypothetical proteins with no reasonable similarity to annotated genes are growing exponentially. It is these genes—this wealth of microbial metabolism, regulation, and other functions—that represent the biggest opportunity for the future of environmental microbiology. What further functions important within biogeochemical cycles might be identified? What roles are currently missing in models of ecosystem services? Sequencing alone will not suffice to identify these hypothetical proteins, meaning that a renewed interest in cultivation methods for metabolic characterization is required.

How best to identify unknown functions within organisms whose obligate interactions with community members preclude isolate cultivation? Enrichment cultures, originally developed by pioneering environmental microbiologist Martinus Beijerinck at the turn of the 19th century, remain a relevant and powerful tool. Enrichment cultures streamline the environmental community to a subset tractable for experimentation. The enriched consortia are frequently more robust than isolate cultures, as beneficial microbial interactions are maintained (9). With interactions intact, previously elusive organisms can be cultivated. The simplified communities within enrichments are also excellent targets for metagenomic sequencing, where high depths of sequence coverage for enriched organisms facilitate genome reconstruction and closure. While enrichment cultures are often expected to be a first step toward isolation of organisms of interest, we argue that current omics approaches can provide equivalent characterization of simplified mixed communities without a need for isolation.

Combinations of metagenome sequencing and enrichment cultivation have already dramatically impacted our understanding of biogeochemical cycles. A major revision to the nitrogen cycle came with the discovery of comammox (10, 11). Both groups that reported *Nitrospira* species capable of conducting complete nitrification used enrichment cultures to simplify an environmental community without the need for isolation. Metagenome sequencing of the enrichments to reconstruct complete or high-quality draft genomes proved cooccurrence of ammonia oxidation genes with the nitrite oxidoreductase for nitrite oxidation (10, 11). Similarly, nitrite-dependent anaerobic methane oxidation (NDAMO) was first reported for a microbial consortium of two organisms: an archaeon and a member of the bacterial candidate phylum NC10 (12). Metagenome sequencing led to a complete genome for “*Candidatus* Methylomirabilis oxyfera,” the first genome for the NC10 phylum (13). Stable isotope probing on the consortia confirmed the unusual intra-aerobic methane metabolism of “*Ca*. Methylomirabilis oxyfera,” where oxygen is produced intracellularly by metabolizing nitrite via nitric oxide into oxygen and dinitrogen gas (13). While the archaeal member of this consortia was eventually lost, the foundational work describing the NDAMO process was conducted on a mixed culture. Similarly, archaeal nitrate-dependent anaerobic methane oxidation was first reported for a member of the ANME-2d lineage from an enriched bioreactor community (14), further proof that isolation is not required for identification of novel microbial biogeochemical processes.

Beyond explorations of biogeochemical cycles, enrichment cultivation coupled with metagenomics can identify interactions present within microbial consortia that contribute to a population’s survival or that support a microbial activity of interest. An enrichment culture developed to examine benzene degradation contains the only known cultivated member of the *Parcubacteria*, a CPR lineage of otherwise-uncultivated phyla (15). Having one member of the *Parcubacteria* in culture will allow experimentation to optimize further cultivation attempts. Enrichment cultures containing *Dehalococcoides mccartyi* sp. have been developed as bioremediation tools for chlorinated solvents, recalcitrant and common groundwater contaminants. Metagenome sequencing of *D. mccartyi* enrichment cultures identified functionally conserved nondechlorinating microbes responsible for maintenance of a reducing environment, provision of cofactors including cobalamin, and carbon turnover (9), activities which improve the biomass yields and dechlorination rates of *D. mccartyi*. Notably, the organisms conducting these roles within different consortia varied, preventing prediction of interactions from taxonomic information alone (9). Enrichment cultures can be effective remediation tools: mechanisms to augment a native microbial community’s ecosystem services with targeted contaminant degradation. Many microbially mediated contaminant transformations remain observed but uncharacterized. Our research group endeavors to address this knowledge gap through meta-omics and enrichment culture experiments, to develop new remediation tools for recalcitrant contaminants.

**FIG 2:**
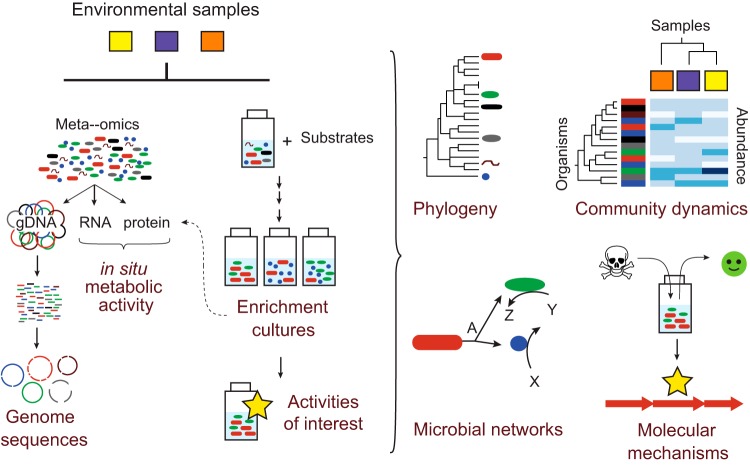
Schematic representation of investigation into microbial diversity, interactions, and metabolic activity using a combined meta-omic and enrichment culture approach. Samples are colored yellow, purple, and orange in the diagram, while the (highly simplified) microbial community is tracked with red, green, and blue (enriched members in culture-based experiments) and black, brown, and gray (environmental members not maintained under the selected enrichment conditions).

Studies connecting in-depth metagenomic characterization of environments with cultivation experiments to confirm predicted functions are the future of environmental microbiology (Fig. 2). Bolstering this approach, metagenome sequencing is now frequently connected to information on RNA, protein, or metabolite expression, thus moving beyond predicted functions to confirmed activities. Importantly, new approaches are needed to enable meaningful interpretations of these large meta-omic data sets across community, population, and molecular levels. Techniques developed for big data analytics in computer science may be applicable, including adapting existing network analysis and statistics tools to biological data. In the coming years, biogeochemical cycles must be revised to take new reactions, partial pathways, and microbial handoffs into account. The microbial “black box” included in ecosystem models will continue to be refined based on community analyses. These revisions will substantially impact models of global biogeochemical cycles and predictions of microbial responses to anthropogenic perturbations.

## References

[1] Venter JC, Remington K, Heidelberg JF, Halpern AL, Rusch D, Eisen JA, Wu D, Paulsen I, Nelson KE, Nelson W, Fouts DE, Levy S, Knap AH, Lomas MW, Nealson K, White O, Peterson J, Hoffman J, Parsons R, Baden-Tillson H, Pfannkoch C, Rogers YH, Smith HO 2004 Environmental genome shotgun sequencing of the Sargasso Sea. Science 304:66–74. doi:10.1126/science.1093857. 15001713

[2] Tyson GW, Chapman J, Hugenholtz P, Allen EE, Ram RJ, Richardson PM, Solovyev VV, Rubin EM, Rokhsar DS, Banfield JF 2004 Community structure and metabolism through reconstruction of microbial genomes from the environment. Nature 428:37–43. doi:10.1038/nature02340. 14961025

[3] Anantharaman K, Brown CT, Hug LA, Sharon I, Castelle CJ, Probst AJ, Thomas BC, Singh A, Wilkins MJ, Karaoz U, Brodie EL, Williams KH, Hubbard SS, Banfield JF 2016 Thousands of microbial genomes shed light on interconnected biogeochemical processes in an aquifer system. Nat Commun 7:13219. doi:10.1038/ncomms13219. 27774985 PMC5079060

[4] Parks DH, Rinke C, Chuvochina M, Chaumeil PA, Woodcroft BJ, Evans PN, Hugenholtz P, Tyson GW 2017 Recovery of nearly 8,000 metagenome-assembled genomes substantially expands the tree of life. Nat Microbiol 2:1533–1542. doi:10.1038/s41564-017-0012-7. 28894102

[5] Hug LA, Baker BJ, Anantharaman K, Brown CT, Probst AJ, Castelle CJ, Butterfield CN, Hernsdorf AW, Amano Y, Ise K, Suzuki Y, Dudek N, Relman DA, Finstad KM, Amundson R, Thomas BC, Banfield JF 2016 A new view of the tree of life. Nat Microbiol 1:16048. doi:10.1038/nmicrobiol.2016.48. 27572647

[6] Brown CT, Hug LA, Thomas BC, Sharon I, Castelle CJ, Singh A, Wilkins MJ, Wrighton KC, Williams KH, Banfield JF 2015 Unusual biology across a group comprising more than 15% of domain Bacteria. Nature 523:208–211. doi:10.1038/nature14486. 26083755

[7] Castelle CJ, Wrighton KC, Thomas BC, Hug LA, Brown CT, Wilkins MJ, Frischkorn KR, Tringe SG, Singh A, Markillie LM, Taylor RC, Williams KH, Banfield JF 2015 Genomic expansion of domain archaea highlights roles for organisms from new phyla in anaerobic carbon cycling. Curr Biol 25:690–701. doi:10.1016/j.cub.2015.01.014. 25702576

[8] Graf DRH, Jones CM, Hallin S 2014 Intergenomic comparisons highlight modularity of the denitrification pathway and underpin the importance of community structure for N_2_O emissions. PLoS One 9:e114118. doi:10.1371/journal.pone.0114118. 25436772 PMC4250227

[9] Hug LA, Beiko RG, Rowe AR, Richardson RE, Edwards EA 2012 Comparative metagenomics of three *Dehalococcoides*-containing enrichment cultures: the role of the non-dechlorinating community. BMC Genomics 13:327. doi:10.1186/1471-2164-13-327. 22823523 PMC3475024

[10] van Kessel MAHJ, Speth DR, Albertsen M, Nielsen PH, Op den Camp HJM, Kartal B, Jetten MSM, Lücker S 2015 Complete nitrification by a single microorganism. Nature 528:555–559. doi:10.1038/nature16459. 26610025 PMC4878690

[11] Daims H, Lebedeva EV, Pjevac P, Han P, Herbold C, Albertsen M, Jehmlich N, Palatinszky M, Vierheilig J, Bulaev A, Kirkegaard RH, von Bergen M, Rattei T, Bendinger B, Nielsen PH, Wagner M 2015 Complete nitrification by *Nitrospira* bacteria. Nature 528:504–509. doi:10.1038/nature16461. 26610024 PMC5152751

[12] Raghoebarsing AA, Pol A, van de Pas-Schoonen KT, Smolders AJP, Ettwig KF, Rijpstra WIC, Schouten S, Damsté JSS, Op den Camp HJM, Jetten MSM, Strous M 2006 A microbial consortium couples anaerobic methane oxidation to denitrification. Nature 440:918–921. doi:10.1038/nature04617. 16612380

[13] Ettwig KF, Butler MK, Le Paslier D, Pelletier E, Mangenot S, Kuypers MMM, Schreiber F, Dutilh BE, Zedelius J, de Beer D, Gloerich J, Wessels HJCT, van Alen T, Luesken F, Wu ML, van de Pas-Schoonen KT, Op den Camp HJM, Janssen-Megens EM, Francoijs KJ, Stunnenberg H, Weissenbach J, Jetten MSM, Strous M 2010 Nitrite-driven anaerobic methane oxidation by oxygenic bacteria. Nature 464:543–548. doi:10.1038/nature08883. 20336137

[14] Haroon MF, Hu S, Shi Y, Imelfort M, Keller J, Hugenholtz P, Yuan Z, Tyson GW 2013 Anaerobic oxidation of methane coupled to nitrate reduction in a novel archaeal lineage. Nature 500:567–570. doi:10.1038/nature12375. 23892779

[15] Luo F, Devine CE, Edwards EA 2016 Cultivating microbial dark matter in benzene-degrading methanogenic consortia. Environ Microbiol 18:2923–2936. doi:10.1111/1462-2920.13121. 26549712

